# Effects of Endometriosis on Anti-Müllerian Hormone

**DOI:** 10.3390/jcm14134495

**Published:** 2025-06-25

**Authors:** Yun Soo Chung, Euna Choi, Jin Kyung Baek, Heeyon Kim, Bo Hyon Yun

**Affiliations:** Department of Obstetrics and Gynecology, Yonsei University College of Medicine, Seoul 03722, Republic of Korea; espera88@yuhs.ac (Y.S.C.); euna_ya@yuhs.ac (E.C.); nupy90@yuhs.ac (J.K.B.); kimhy@yuhs.ac (H.K.)

**Keywords:** endometriosis, anti-Müllerian hormone, fertility, ovarian reserve

## Abstract

**Background/Objectives**: As the population of women with endometriosis increases, approximately 10% of those of reproductive age experience symptoms such as pelvic pain, painful menstruation, and infertility. Individuals with endometriosis usually undergo multiple surgeries due to the high recurrence rate of the condition. However, ovarian surgery tends to reduce the ovarian reserve, presenting a dilemma when deciding whether to recommend surgery or medical treatment for women of reproductive age. The impact of endometriomas on the residual volume of ovarian tissue remains controversial, and it is unclear whether endometriosis itself or endometriomas are the primary problem. In this study, we aimed to investigate whether women with endometriosis have lower levels of anti-Müllerian hormone than women with healthy ovaries before treatment initiation. **Methods**: A total of 298 participants enrolled in the endometriosis cohort at Severance Hospital, Korea, from 1 October 2020 to 1 July 2024 were included in this study. Of these, 63 participants were from a retrospective study, and 235 were from a prospective study. Due to the use of different assay methods between the reference values and anti-Müllerian hormone measurements from Severance Hospital, a correction was applied using the regression equation. The mean anti-Müllerian hormone levels for individuals with endometriosis were corrected with the regression equation and compared to those of the reference group for each age group using a one-sample *t*-test. **Results**: Anti-Müllerian hormone levels decreased with age in the endometriosis group. When comparing mean anti-Müllerian hormone concentrations between the endometriosis group and reference values, among 168 participants aged 20–31 years, the corrected mean anti-Müllerian hormone concentration was 5.96 ± 3.22 ng/mL, higher than the reference value of 4.94 ± 0.17 ng/mL (*p* < 0.01). Among 31 participants aged 35–37 years, the corrected average anti-Müllerian hormone value was 4.33 ± 3.06 ng/mL, compared to the reference anti-Müllerian hormone level of 3.22 ± 0.15 ng/mL (*p* = 0.05). There were no significant differences in corrected anti-Müllerian hormone levels between the 32–34-, 38–40-, 41–43-, and ≥44 years age groups. **Conclusions**: Patients with endometriosis, especially those aged 20–31 years, tended to have higher anti-Müllerian hormone levels than did individuals with healthy ovaries. In other age groups, there were no differences. Given that these levels do not differ significantly across age groups, it is difficult to conclude that patients with endometriosis have a reduced ovarian reserve.

## 1. Introduction

The incidence of endometriosis is unclear because pathological diagnosis is challenging [1]. Despite difficulties in diagnosing endometriosis, its prevalence is increasing [2,3,4]. Approximately 10% of women of reproductive age are affected by endometriosis-related subfertility [4,5,6]. Younger women with severe endometriosis experience pelvic pain and dysmenorrhea [7]; furthermore, they often experience impaired ovarian reserve and diminished oocyte yield [8]. Previous reports have demonstrated that diminished ovarian reserves resulted in decreased anti-Müllerian hormone (AMH) levels in patients with ovarian endometriosis [9,10]. On the other hand, some reported that endometriosis does not affect ovarian reserve and that there is no significant difference in AMH levels between individuals with endometriosis and those with normal ovaries [5].

As women’s social and professional statuses increase, childbearing is increasingly delayed, compared to the past. Consequently, there is growing concern about ovarian reserve, biomarkers predicting ovarian function, and whether endometriosis affects ovarian reserve. Approximately 40–50% of patients experience spontaneous recurrence of endometriosis within 5 years following surgery [11,12,13]. Therefore, physicians must carefully consider the balance between surgical and medical treatments when treating women of childbearing age. If individuals with endometriosis have lower baseline AMH levels due to a reduced ovarian reserve, opting for surgical treatment may pose additional challenges for physicians.

Several biomarkers are used to predict ovarian reserve, including follicle-stimulating hormone (FSH), AMH, basal estradiol, and antral follicle counts [14]. AMH, a member of the transforming growth factor-beta superfamily, is a homodimeric disulfide-linked glycoprotein [15,16]. It is produced by the granulosa cells of growing follicles from the primary to the small antral stage [5,17]. AMH expression increases in follicles up to 8 mm in size; however, it is absent in follicles larger than 8 mm [17]. Among the various biomarkers for predicting ovarian reserve, AMH is particularly useful because it can be measured at any point in the menstrual cycle [5,18]. In this study AMH served as a biomarker for evaluating ovarian reserve.

According to previous research, different AMH assays can yield varying results in AMH levels [17,19]. Therefore, when interpreting AMH values obtained from different assay platforms, appropriate correction methods should be applied [17,19].

AMH levels have been reported to be lower in women with endometriosis compared to those with benign conditions or healthy ovaries [9,10], and the decline in AMH appears to occur more rapidly in these individuals [20]. Regardless of the location of endometriosis, lower AMH levels have been observed among infertile women [21]. Additionally, some studies have reported that endometriosis is associated with a reduced antral follicle count and AMH levels, along with increased FSH-markers that are all indicative of diminished ovarian reserve [22]. However, since there are no precise measurements for ovarian reserve, assuming that a low AMH level in women with endometriosis indicates reduced ovarian reserve remains controversial [23]. Some studies have reported that endometriosis alone may not be a cause of decreased AMH levels [5,24]. There are reference serum AMH values established for Korean women with regular menstruation [16]. Different studies have reported varying correlations between endometriosis and AMH levels, highlighting the controversy surrounding this topic. Therefore, the aim of this study was to investigate whether women with endometriosis exhibit characteristic AMH levels compared to reference values. We also sought to explore the potential of AMH as a biomarker to assist physicians in interpreting AMH levels in patients with endometriosis, and to help guide decisions regarding medical or surgical treatment and considerations for fertility preservation.

## 2. Materials and Methods

### 2.1. Study Participants and Data Collection

A total of 298 participants were enrolled in the endometriosis cohort at Severance Hospital in Seoul, Korea, from 1 October 2020 to 1 July 2024. This study combined prospective and retrospective cohort studies. A total of 63 participants diagnosed with endometriosis before 15 October 2020 were included in the retrospective cohort, while 235 participants diagnosed after 15 October 2020 were included in the prospective cohort.

**Figure 1:**
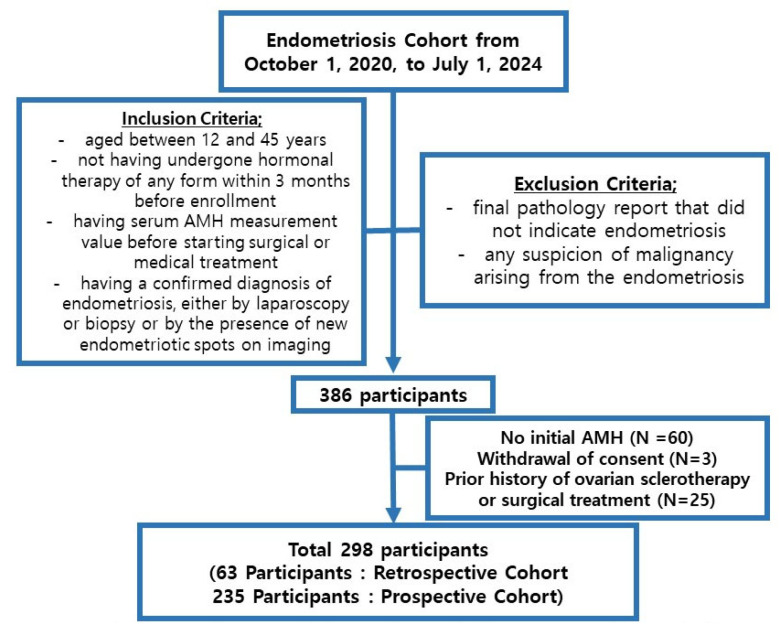
Diagram of the endometriosis group study population.

The inclusion and exclusion criteria for both cohorts were as follows (Figure 1):

Inclusion criteria: being aged between 12 and 45 years; not having undergone hormonal therapy of any form within 3 months before enrollment; having a serum AMH measurement value before starting surgical or medical treatment; having a confirmed diagnosis of endometriosis, either by laparoscopy or biopsy or by the presence of new endometriotic spots on imaging (such as pelvic computed tomography or pelvic magnetic resonance imaging).

The exclusion criterion was a final pathology report that did not indicate endometriosis or any suspicion of malignancy arising from the endometriosis.

For the prospective cohort, the Endometriosis Health Profile Questionnaire was administered at enrollment and every 6 months thereafter for up to 5 years. The CA-125 and AMH tests were conducted before treatment initiation. A total of 60 participants had missing information, 3 participants withdrew consent, and 25 participants had a history of either sclerotherapy or ovarian surgery. These participants were excluded from the analysis.

For both cohorts, follow-up pelvic sonography, serum AMH, and CA-125 tests were conducted every 6–12 months for 5 years. After 5 years, assessment of CA-125 level and sonography were repeated based on endometriosis-related symptoms; this was performed every 6–24 months.

### 2.2. AMH Assay

AMH levels were measured in blood samples collected by venipuncture using the cobas e801 modules (Roche Diagnostics International AG; Rotkreuz, Switzerland) through a two-step electrochemiluminescence immunoassay. This assay employs the sandwich principle to detect biotinylated monoclonal AMH-specific antibodies. During the first incubation, a sandwich complex is formed between the sample (30 μL), biotinylated monoclonal AMH-specific antibody, and biotinylated monoclonal AMH-specific antibody tagged with a ruthenium complex. In the second incubation, streptavidin-coated microparticles are added, allowing the sandwich complex formed during the first incubation to bind to the solid phase through interactions between streptavidin and biotin. This complex is then absorbed into the measuring cell and attached to the electrode. When voltage is applied to the electrodes, chemiluminescence is emitted, and this signal is detected using a photomultiplier. This assay method was calibrated and standardized against the Beckman Coulter AMH Gen II ELISA (Brea, CA, USA).

The measurement range for AMH is from 0.01 to 23 ng/mL. AMH levels below 0.01 ng/mL were reported as <0.01 ng/mL, and levels above 23 ng/mL were reported as >23 ng/mL. The blank had a value of 0.007 ng/mL, the limit of detection was 0.01 ng/mL, and the limit of quantitation was 0.03 ng/mL.

Precision and comparison tests between the Beckman Coulter array (Brea, CA, USA) and the cobas e801 modules (Roche Diagnostics International AG; Rotkreuz, Switzerland) were conducted by the Department of Laboratory Medicine at Severance Hospital. For correction, the following regression equation was applied:

Mean Beckman Coulter = (Mean Cobas (ng/mL) − 0.1618)/0.6691 (R^2^ = 1)

### 2.3. Reference AMH

We adopted reference age-specific serum AMH levels from the only study reporting age-specific serum AMH levels conducted by You et al. [16]. The mean, percentiles, and 95% confidence intervals published in the original article were used for the analyses. These results were based on data from 1,298 women aged 20–50 years who had regular menstrual cycles [16]. This group is the control group and inclusion criteria is women with regular menstrual cycle intervals of 21–35 days and aged between 20 and 50 years. Exclusion criteria were women with polycystic ovary syndrome, a history of ovarian surgery, a body mass index > 30 kg/m^2^, or any endocrine diseases, such as thyroid disease, diabetes mellitus, or Cushing’s syndrome. The reference AMH values were analyzed using the Immunotech ELISA from Beckman Coulter (Brea, CA, USA).

### 2.4. Statistical Analysis

The mean AMH levels in individuals with endometriosis were calculated for each age group. The collected AMH levels followed a normal distribution, and a one-sample *t*-test was performed to determine whether the mean AMH level in the endometriosis group differed from the reference value. Pearson correlation analysis was used to assess the relationship between age and AMH levels in patients with endometriosis. Percentile values of AMH levels were compared between the endometriosis and healthy ovarian groups. The bootstrap method was used to compare median values. If the reference median falls within the 95% bootstrap confidence interval, it indicates no statistically significant difference between the groups. All statistical analyses were conducted using IBM SPSS Statistics for Windows version 28 (IBM Corp., Armonk, NY, USA) and R version 4.4.1 (The R Foundation, www.R-project.org). Statistical analyses and visualization were performed using Microsoft Excel version 16 (Microsoft Corp., Redmond, WA, USA) and R version 4.4.1 (The R Foundation, www.R-project.org). Statistical significance was set at a two-sided *p*-value < 0.05.

### 2.5. Ethics Statement

Ethical approval for data collection was obtained from the ethics committee of our institutional review board (4-2024-0787), and all participants provided written informed consent.

## 3. Results

### 3.1. Mean Age and Basal Characteristics

The participant characteristics of each age group are presented in Table 1. The different age groups displayed varying treatment percentages. Those who were younger tended to have a higher percentage of sclerotherapy, while those who were of an older age tended to undergo more surgeries (Table 1). A small percentage of individuals in each age group were treated for peritoneal endometriosis, while a higher percentage had a unilateral ovary (Table 1). There was no history of endometriosis recurrence, as participants with a history of surgical or sclerotherapy treatments were excluded (Table 1).

Participants were stratified by age, and the means of the age groups were compared, as shown in Table 2. There were no significant age differences among the age groups: 32–34, 35–37, 38–40, and 41–43 years. The mean age of the 20–31 year group was 26.9 ± 3.1 years, compared to 29.1 ± 0.1 years for the reference group (*p* < 0.01). In the age group ≥ 44 years, the mean age for the reference group was 45.6 ± 0.3 years, while it was 44.7 ± 0.5 years for the endometriosis group (*p* < 0.01).

### 3.2. Mean AMH of Each Age Group

**Figure 2:**
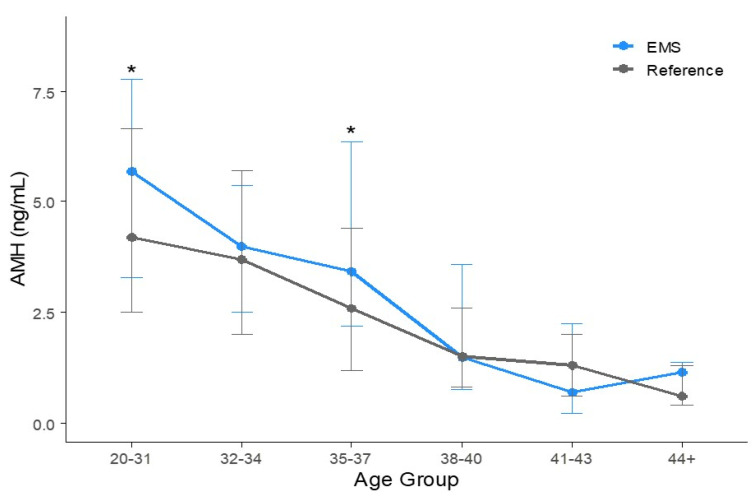
Age-specific comparison of AMH levels between EMS and reference groups using median and interquartile range (IQR). Vertical error bars represent the 25th and 75th percentiles. Asterisks (*) indicate statistically significant differences in the median based on bootstrap 95% confidence intervals.

A trend of decreasing AMH levels with age was observed in the endometriosis group, as shown in Supplemental Figure S1. When comparing the mean AMH concentrations between the endometriosis group and reference values, not all endometriosis groups exhibited significantly different AMH levels compared to the reference group (Figure 2). Among 168 participants aged 20–31 years, the corrected mean AMH concentration was 5.96 ± 3.22 ng/mL, which is slightly higher than the reference value of 4.94 ± 0.17 ng/mL (*p* < 0.01). There were 30 participants aged between 32 and 34 years, and the corrected average AMH value was 4.49 ± 3.33 ng/mL for them, compared to the reference AMH value of 4.25 ± 0.17 ng/mL (*p* = 0.70). The corrected mean AMH concentration of 31 participants aged 35–37 years was 4.33 ± 3.06 ng/mL, whereas the reference AMH level was 3.22 ± 0.15 ng/mL (*p* = 0.05). Among 34 participants aged 38–40 years, the corrected average AMH value was 2.51 ± 2.58 ng/mL, compared to the reference value of 2.13 ± 0.15 ng/mL (*p* = 0.39). The corrected average AMH level for 26 participants aged 41–43 years was 1.33 ± 1.43 ng/mL, while the reference value was 1.47 ± 0.13 ng/mL (*p* = 0.62). Among nine participants aged over 44 years, the corrected mean AMH concentration was 0.93 ± 0.68 ng/mL, compared to the reference value of 0.95 ± 0.14 ng/mL (*p* = 0.94; Table 2).

### 3.3. Percentile AMH Concentration of Each Age Group

**Figure 3:**
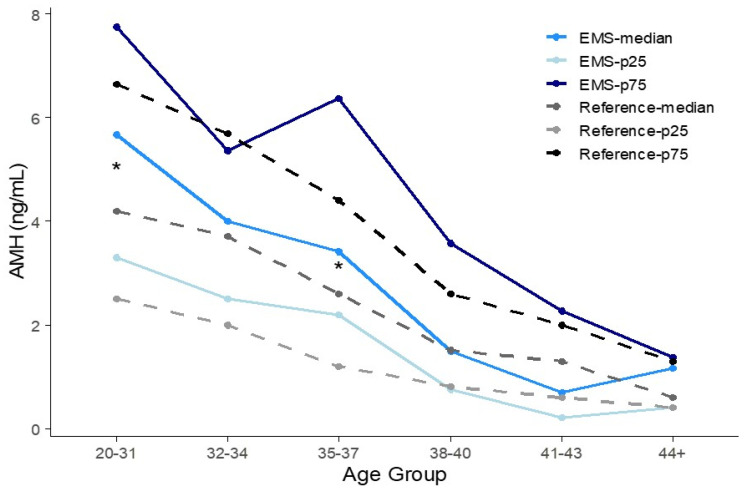
AMH percentiles (25th, median, 75th) plotted by age group for EMS and reference populations. Each percentile is shown as an independent line. Asterisks (*) mark significant differences in median values identified by bootstrap analysis.

The 25th, 50th, and 75th percentile values of serum AMH levels were compared with the reference intervals. In Figure 2, the 25th–75th percentiles are represented as the vertical error bars. The median and interquartile ranges of the reference and EMS groups are compared in Table 2 and illustrated in Figure 2. The percentiles of AMH concentrations for the reference and the corrected percentiles of AMH concentrations for the endometriosis group are presented in Figure 3. As age increases, AMH levels exhibit a decreasing trend. However, it is difficult to consistently determine whether the reference values or those of the corrected endometriosis group are higher. As shown in Table 2 and Figure 2 and Figure 3, according to the bootstrap method, the 20–31 and 35–37-year-old age group showed statistically significant differences in percentile values between the control and EMS groups.

## 4. Discussion

As the age at marriage increases and the use of assisted reproductive technology grows, serum AMH levels become crucial in assessing female fertility [25,26,27]. AMH is produced by the granulosa cells of growing follicles up to 8 mm in size [17]. Given that AMH is secreted by small antral follicles that are independent of gonadotropins, its levels remain stable throughout both intra- and inter-cycle variations [28]. This stability is one reason why AMH is widely used to measure the ovarian reserve among various biomarkers.

Women are born with a finite number of primordial follicles, and by the age of 30 years, the number of these primordial follicles begins to decline, leading to reduced ovarian reserve [25]. This decrease in follicle count contributes to an age-related decline in AMH levels [29]. According to our study, AMH levels decrease with age; however, women with endometriosis do not have lower AMH levels compared to those with normal ovaries. This finding is not consistent with some previous studies [9,10]. In contrast, some studies have reported that women with endometriotic ovarian cysts have significantly lower AMH levels compared to those with other benign cysts [30]. Patients with endometriosis have been reported to have low preoperative AMH levels [5,31,32]. Endometriosis, characterized by an estrogen-dependent chronic inflammatory status, leads to notable alterations in both the function and structure of the ovarian follicles [23,33]. Granulosa cells in endometriotic cysts are impacted by oxidative stress, inflammation, disrupted energy metabolism, apoptosis, and inappropriate steroidogenesis [34,35]. Estrogen promotes proliferation, invasion, and self-estrogen production in the endometrial stromal cells of ectopic lesions [35]. It also stimulates the production of pro-inflammatory factors, and long-term exposure increases the level of transforming growth factor beta-1, contributing to fibrotic tissue formation [35,36,37,38]. Increased apoptosis of granulosa cells during endometriosis is associated with disruptions in the cell cycle [39,40]. Previous studies have indicated that apoptotic bodies resulting from this process reduce oocyte recovery time and increase the number of empty follicles [35]. These inflammatory, fibrotic, apoptotic, and oxidative stresses associated with endometriosis negatively affect the ovarian reserve [35].

However, some previous studies have reported that individuals with endometriosis do not have lower AMH levels compared to controls [5,24,41,42]. The control group consisted of age-matched women with no known or suspected endometriosis, based on medical history and ultrasonography. Some studies found no significant difference in AMH levels between individuals with endometriosis and controls, suggesting that AMH alone may not be a reliable predictor of ovarian reserve [41]. Others have reported that endometriosis itself does not affect oocyte quality [42]. One study evaluated follicular IL-6 levels, which are associated with inflammatory responses, and reported that despite elevated IL-6 levels, the follicular AMH levels in the endometriosis group were not lower than those in the control group [42]. Another study reported that a reduction in AMH levels is associated only with a history of endometrioma surgery, rather than with the presence of endometriosis itself [24].

In the current study, although the mean AMH concentrations were lower in the 20–31-year-old control group, no statistically significant differences were observed in the other age groups. This might be related to age, as the average age of the EMS group within the 20–31 age range was younger than that of the control group. The lack of significance may be due to the small sample size and the use of different AMH assay methods. Given that AMH levels naturally decline with age, the findings of this study do not support the conclusion that individuals with endometriosis have lower AMH levels than those without the condition.

This study had some limitations. Despite the higher prevalence of endometriosis in Asia, this study had small sample sizes in certain age groups. Due to the limited sample size, although the mean AMH level in the endometriosis group was lower, the *p*-value did not reach significance. However, by including both prospective and retrospective cohort participants, this study aimed to increase the sample size and enhance statistical power. Analyzing prospective and retrospective data together can introduce challenges such as selection bias, information bias, and statistical handling issues due to differences in enrollment timing. To address this, participants with missing AMH values were excluded from the analysis. Additionally, since AMH levels are not influenced by the timing of enrollment, combining data from both cohorts did not compromise the accuracy of the analysis. Another limitation of this study is that the reference AMH levels and the AMH levels in the endometriosis group were measured using different assays. Although a regression equation based on precision and comparison testing was applied to minimize bias, this discrepancy may still have introduced variability. Generalization bias may arise depending on the sample size used to develop the regression equation. Therefore, future studies should be conducted with larger sample sizes using the same AMH assay to ensure consistency and accuracy. Another limitation of this study is that the reference AMH levels were obtained from previously published research, in which the inclusion and exclusion criteria were not clearly defined. Since the purpose of that study was to establish age-related reference values, we assumed that participants did not have any gynecological conditions. However, it remains unclear whether individuals with ovarian cysts or those using oral contraceptives were included, which introduces potential variability in the reference data. The strength of this study was that it compared the AMH levels of Korean women with endometriosis with the reference AMH values of women with healthy ovaries. Since the prevalence of endometriosis is higher in Asia compared to that in other ethnicities [43], the results of this study will contribute to the understanding of the nature of endometriosis in relation to the ovarian reserve.

## 5. Conclusions

As women age, AMH concentrations decrease, regardless of whether they have healthy ovaries or ovaries affected by endometriosis. Compared with individuals with healthy ovaries, those with endometriosis did not show significantly lower AMH values. Since AMH levels in the endometriosis group remain controversial, further studies with larger sample sizes and standardized AMH assays are needed.

## Figures and Tables

**Table 1:** Participant characteristics of each age group in the study population.

Age (Yr)		20–31	32–34	35–37	38–40	41–43	≥44
**N**		168	30	31	34	26	9
**Average Age (yr)**		26.85	32.80	36.13	38.92	41.81	44.67
**Initial treatment after the diagnosis**	**Sclerotherapy (%)**	80 (47.6)	15 (50)	13 (41.9)	17 (50)	11 (42.3)	1 (11.1)
	**Operation d/t endometrioma (%)**	50 (29.8)	5 (16.7)	10 (32.3)	15 (44.1)	15 (57.7)	8 (88.9)
	**Medication (%)**	36 (21.4)	10 (33.3)	7 (22.6)	2 (5.9)		
	**Operation d/t EMS other than ovary (%)**	2 (1.2)	0	1 (3.2)			
**Tracking loss after initial visit (%)**				1 (3.2)			
**Endometriosis**	**Peritoneum (%)**	4 (2.4)	2 (6.7)	1 (2.8)	0 (0)	0 (0)	1 (8.3)
	**Unilateral ovary (%)**	114 (67.9)	18 (60)	26 (80.6)	21 (64.1)	19 (68.8)	5 (58.3)
	**Bilateral ovary (%)**	50 (29.8)	10 (33.3)	4 (16.7)	13 (35.9)	7 (31.3)	3 (33.3)
**Recurrence of endometriosis** (%)		0(0)	0 (0)	0 (0)	0 (0)	0 (0)	0 (0)
**Previous treatment of endometriosis**	**Surgery (%)**	0 (0)	0 (0)	0 (0)	0 (0)	0 (0)	0 (0)
	**Medication (%)**	11 (6.5)	4 (13.3)	2 (6.5)	2 (5.9)	2 (7.7)	0 (0)
	**None (%)**	157 (93.5)	26 (86.7)	29 (93.5)	32 (94.1)	24 (92.3)	9 (100)
	**Sclerotherapy (%)**	0 (0)	0 (0)	0 (0)	0 (0)	0 (0)	0 (0)

N: number; EMS: endometriosis; Yr: years; d/t: due to.

**Table 2:** Comparison of age and AMH levels between the reference group and EMS groups.

Age (yr)	N		Age (yr) Mean ± SD			AMH (ng/mL) Mean ± SD (95% CI)			25th Percentile		50th Percentile (Median)		75th Percentile		Bootstrap 95% CI
	**Ref.**	**EMS**	**Ref.**	**EMS**	***p*-Value**	**Ref.**	EMS *	***p*-Value**	**Ref.**	EMS * (N)	**Ref.**	EMS * (N)	**Ref.**	EMS * (N)	EMS *
20–31	377	168	29.1 ± 0.1	26.9 ± 3.1	**<0.01**	4.94 ± 0.17 (4.61–5.25)	5.96 ± 3.22 (5.47–6.44)	**<0.01**	2.5	3.29(24)	4.2	5.68 (57)	6.65	7.75 (108)	**5.14–6.05**
32–34	331	30	33.1 ± 0.4	32.8 ± 0.8	0.06	4.25 ± 0.17 (3.92–4.57)	4.49 ± 3.33 (3.30–5.68)	0.70	2.00	2.50 (5)	3.70	4.00 (13)	5.70	5.36 (23)	2.85–4.56
35–37	283	31	35.9 ± 0.5	36.1 ± 0.8	0.85	3.22 ± 0.15 (2.92–3.51)	4.33 ± 3.06 (3.25–5.40)	0.05	1.20	2.19 (2)	2.60	3.42 (9)	4.40	6.37 (17)	**2.70–5.08**
38–40	173	34	39.0 ± 0.6	38.9 ± 0.8	0.94	2.13 ± 0.15 (1.83–2.44)	2.51 ± 2.58 (1.65–3.38)	0.39	0.80	0.76 (9)	1.50	1.49 (17)	2.60	3.58 (24)	1.13–2.28
41–43	87	26	41.7 ± 0.1	41.8 ± 0.8	0.52	1.47 ± 0.13 (1.21–1.71)	1.33 ± 1.43 (0.78–1.88)	0.62	0.60	0.21 (12)	1.30	0.70 (15)	2.00	2.26 (17)	0.34–2.06
≥44	47	9	45.6 ± 0.3	44.7 ± 0.5	<**0.01**	0.95 ± 0.14 (0.68–1.23)	0.93 ± 0.68 (0.49–1.37)	0.94	0.40	0.40 (2)	0.60	1.15 (3)	1.30	1.37 (6)	0.13–1.67

AMH: anti-Müllerian hormone; N: number; Ref: Reference; EMS: endometriosis; SD: standard deviation; CI: confidence interval; Yr: year. Data presented in bold indicates statistical significance. * AMH values were corrected with the regression equation: (Mean Cobas (ng/mL) − 0.1618)/0.6691 = Mean Beckman Coulter (R^2^ = 1).

## Data Availability

The raw data supporting the conclusions of this article will be made available by the authors upon request.

## Supplementary Materials

The following supporting information can be downloaded at: https://www.mdpi.com/article/10.3390/jcm14134495/s1, Figure S1: Age-specific anti-müllerian hormone (AMH) in the endometriosis group; Figure S2: Anti-müllerian hormone (AMH) concentration of in the endometriosis group The 25th–75th percentiles are represented as boxes, with the median indicated by the midline. The maximum and minimum values are shown as bars extending above and below the box.
