# Supplementary figures and images for: Effects of Endometriosis on Anti-Müllerian Hormone
Source: J Clin Med. 2025 Jun 25;14(13):4495. doi: 10.3390/jcm14134495 (PMC12249931; doi:10.3390/jcm14134495)

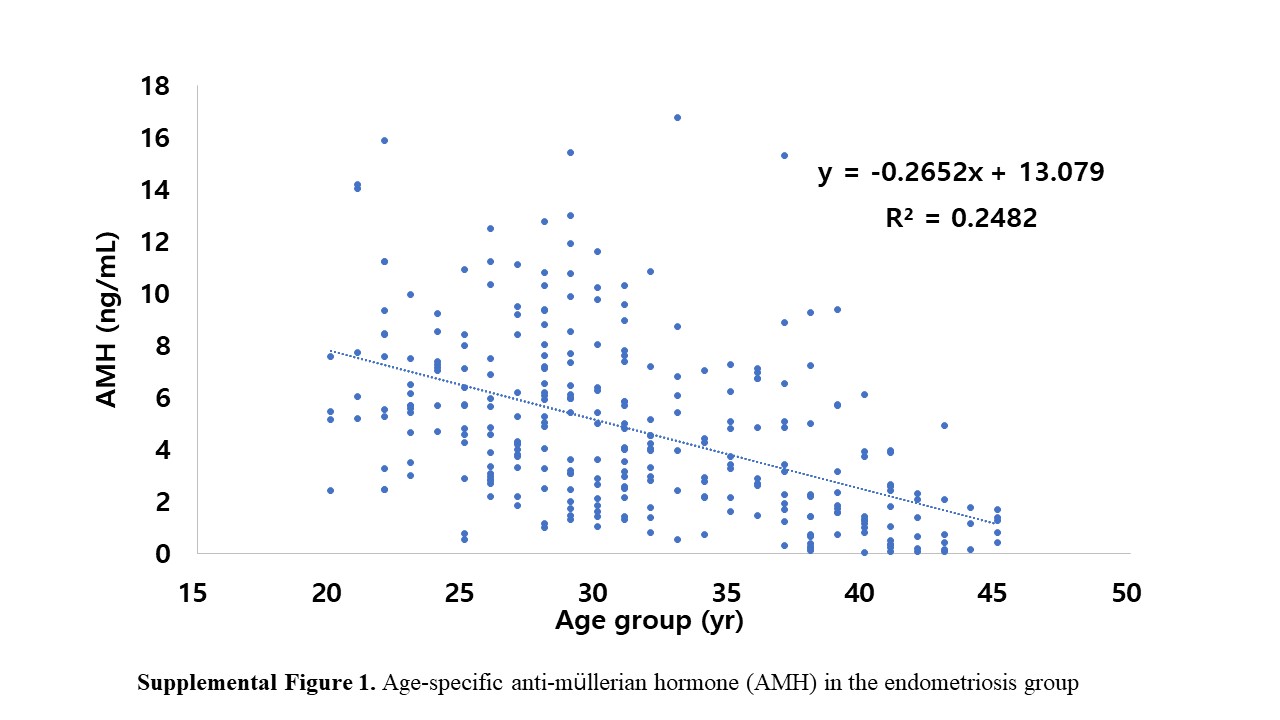

Supplement: Supplementary file 1 [file jcm-14-04495-s001.zip › Supplemental Figure S1.jpg]

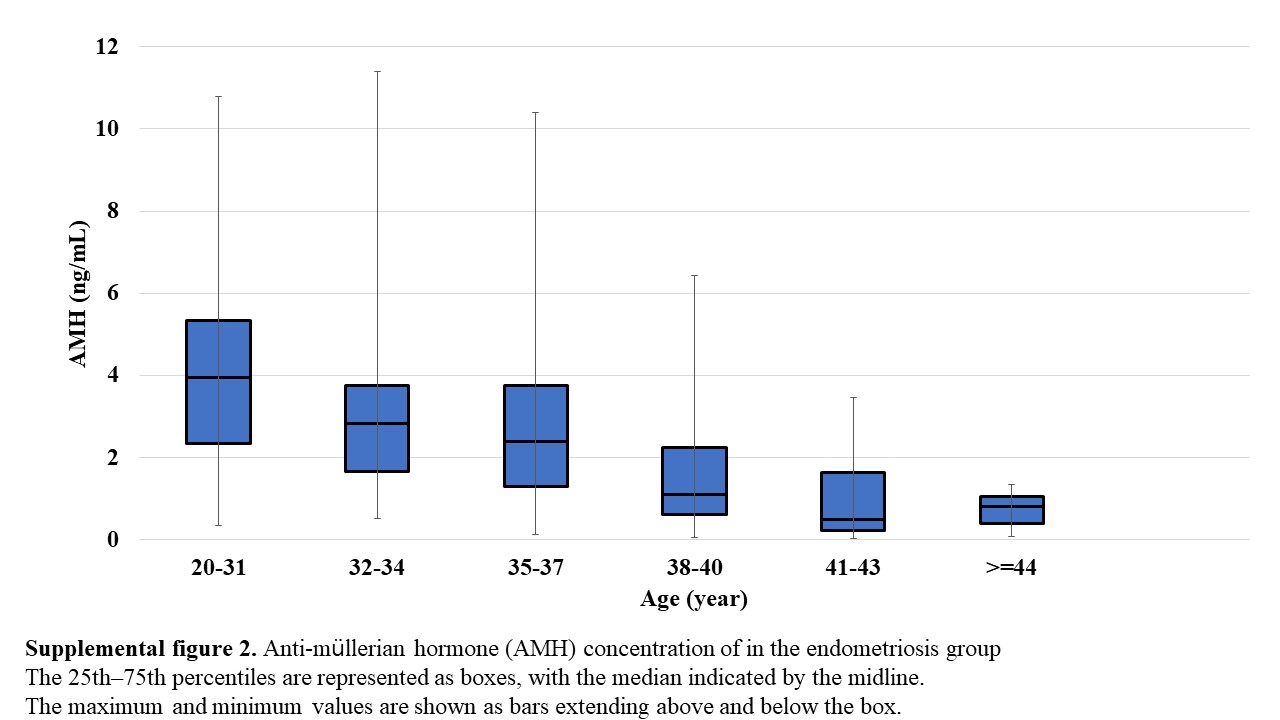

Supplement: Supplementary file 1 [file jcm-14-04495-s001.zip › Supplemental Figure S2.jpg]
